# Radiation Monotherapy for Primary Cutaneous Diffuse Large B-cell Lymphoma, Leg Type: A Case Report

**DOI:** 10.7759/cureus.114059

**Published:** 2026-08-06

**Authors:** Maudood Rana, Danielle Jamison, L. Jeffrey Medeiros, Ahmed Taher, Haleigh Mistry

**Affiliations:** 1 Department of Neuroscience, Brown University, Providence, USA; 2 Department of Lymphoma and Myeloma, The University of Texas MD Anderson Cancer Center, Houston, USA; 3 Department of Hematopathology, The University of Texas MD Anderson Cancer Center, Houston, USA; 4 Department of Diagnostic Imaging, The University of Texas MD Anderson Cancer Center, Houston, USA; 5 Department of Pediatrics, The University of Texas MD Anderson Cancer Center, Houston, USA

**Keywords:** elderly, gray, non-hodgkin lymphoma, radiotherapy, skin nodule

## Abstract

Primary cutaneous diffuse large B-cell lymphoma, leg type (PCDLBCL-LT), is a rare and distinct subtype of non-Hodgkin lymphoma. PCDLBCL-LT is characterized by malignant B-cell proliferation within the skin, often presenting as erythematous nodules or plaques primarily affecting the lower extremities. Differentiating PCDLBCL-LT from other primary cutaneous B-cell lymphomas (CBCLs) can be difficult. However, an accurate diagnosis is crucial to identify the appropriate treatment. Once diagnosed, chemotherapy, including rituximab as a monotherapy or combined with doxorubicin, cyclophosphamide, vincristine, and prednisone (R-CHOP), is the standard treatment for PCDLBCL-LT; yet many PCDLBCL-LT patients are elderly and frail, making them poor candidates for chemotherapy. Radiation monotherapy may be a more appropriate treatment for these patients, particularly those with localized lesions, because of its excellent local control, favorable toxicity profile, and overall survival (OS) comparable to chemotherapy alone.

In this report, we present the case of a 93-year-old woman with stage II leg-localized PCDLBCL-LT who declined to receive systemic therapy due to her concerns regarding her age and fragility. The patient achieved a complete metabolic response following a hypofractionated course of radiation monotherapy and remained disease-free at her five-year survival follow-up, supporting the suggestion that radiation monotherapy may be an effective treatment approach for select elderly patients.

## Introduction

Primary cutaneous lymphomas (PCLs) are a diverse group of extranodal non-Hodgkin lymphomas confined to the skin [1]. Approximately 75% of PCLs arise from T cells, with the remaining quarter arising from B cells [1,2]. Among the three most common subtypes of primary cutaneous B-cell lymphomas (CBCLs), primary cutaneous diffuse large B-cell lymphoma, leg type (PCDLBCL-LT), is the most aggressive [1].

PCDLBCL-LT is a rare neoplasm, representing approximately 20% of all CBCLs and less than 5% of all PCLs [2,3]. PCDLBCL-LT has a variable clinical presentation but predominantly affects elderly patients (median age at diagnosis in the 70s) with a strong female predominance (2-4:1) [1,3,4]. The neoplasm typically presents as solitary or clustered nodules or plaques that are ulcerated, rapidly growing, and erythematous, with 10% to 15% of tumors or nodules occurring in non-leg locations [1,4]. Approximately 10% to 20% of patients with PCDLBCL-LT also have extracutaneous lesions, the most common sites of which include lymph nodes, bone marrow, or the central nervous system [5]. Both histopathologic and immunophenotypic features can help distinguish PCDLBCL-LT from other types of CBCL, which is necessary for an accurate diagnosis and, subsequently, the identification of an appropriately aggressive treatment [1,6]. For example, PCDLBCL-LT does not exhibit epidermotropism and typically consists of a monotonous population of centroblasts or immunoblasts, lacking centrocytes [3,5]. Immunophenotypic analysis of PCDLBCL-LT is often positive for B-cell markers, usually positive for BCL2 and MUM1/IRF4, more variably positive for BCL6 and MYC, and negative for CD10, unlike other CBCLs [3,5].

PCDLBCL-LT has the poorest prognosis of the three most prevalent CBCLs [1]. While the addition of rituximab improved survival, the five-year survival rate for PCDLBCL-LT is estimated at 40%-60%, compared with 95%-100% for the other two CBCLs [4,7,8]. Patient age, specifically age older than 75 years, multiple lesions, and extent of tumour dissemination are all negative prognostic factors [3,4,9]. Further, PCDLBCL-LT patients with leg localization have a poorer prognosis than those with other localizations (three-year survival rates of 43% versus 77%, respectively) [3,9].

PCDLBCL-LT is recognized as a distinct entity in the WHO-EORTC and ICC classifications because of its differing histology from and worse prognosis than other CBCL subtypes [1,8,10,11]. Current National Comprehensive Cancer Network (NCCN) guidelines recommend polatuzumab vedotin with rituximab, cyclophosphamide, doxorubicin, and prednisone (Pola-R-CHP), combined with involved-site radiation therapy (ISRT) or clinical trials as first-line therapy for solitary, regional disease; local ISRT alone is suggested for patients who cannot tolerate chemoimmunotherapy [12].

Because PCDLBCL-LT has a median age of diagnosis in the seventies, many patients are fragile or have comorbidities that make intensive chemoimmunotherapy challenging, with many patients foregoing it [3]. As such, local ISRT may represent the only curative-intent treatment for these patients. However, a significant clinical gap exists regarding the efficacy and durability of radiation monotherapy (i.e., local ISRT alone) as the definitive treatment approach for elderly patients who do not undergo chemoimmunotherapy. Radiation monotherapy has demonstrated good local disease control, but its ability to provide durable long-term remission in PCDLBCL-LT is poorly characterized; most data are from small retrospective studies with relatively short follow-up periods [13].

We report this case to highlight the effectiveness of radiation monotherapy as a definitive treatment for PCDLBCL-LT in an elderly patient with several negative prognostic factors, her favorable five-year outcome, and a review of the relevant literature.

## Case presentation

**Figure 1:**
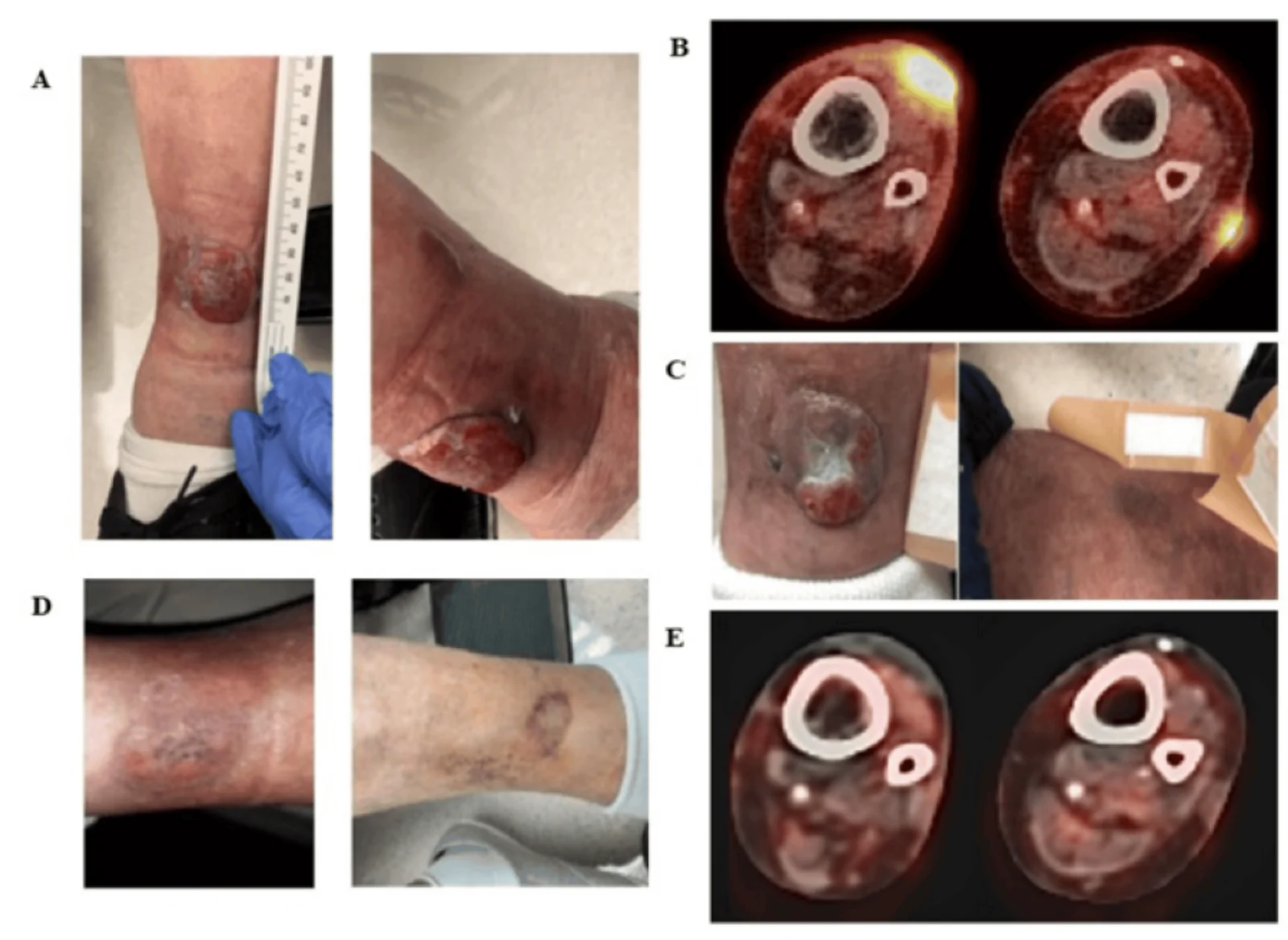
Clinical and imaging progression of the patient’s leg lesion prior to and after radiation monotherapy. (A) The left anterior (left) and left lateral (right) views of the patient’s lesion on her ankle, including the lesion measurement (3.5 cm) in the anterior view. (B) The PET/CT image obtained at diagnosis shows two hypermetabolic cutaneous/subcutaneous lesions on the left distal leg. (C) The lesion prior to initiating radiation therapy. (D) The lesion at seven days (left) and six months (right) after radiation therapy. (E) The PET/CT image obtained three months after radiation therapy demonstrates a complete metabolic response. cm: centimeter; PET/CT: positron emission tomography/computed tomography

We present the case of a 93-year-old female with a past medical history of Alzheimer’s disease and well-controlled hypertension who had a two-week history of a pruritic skin lesion on the left ankle (Figure 1A).

**Figure 2:**
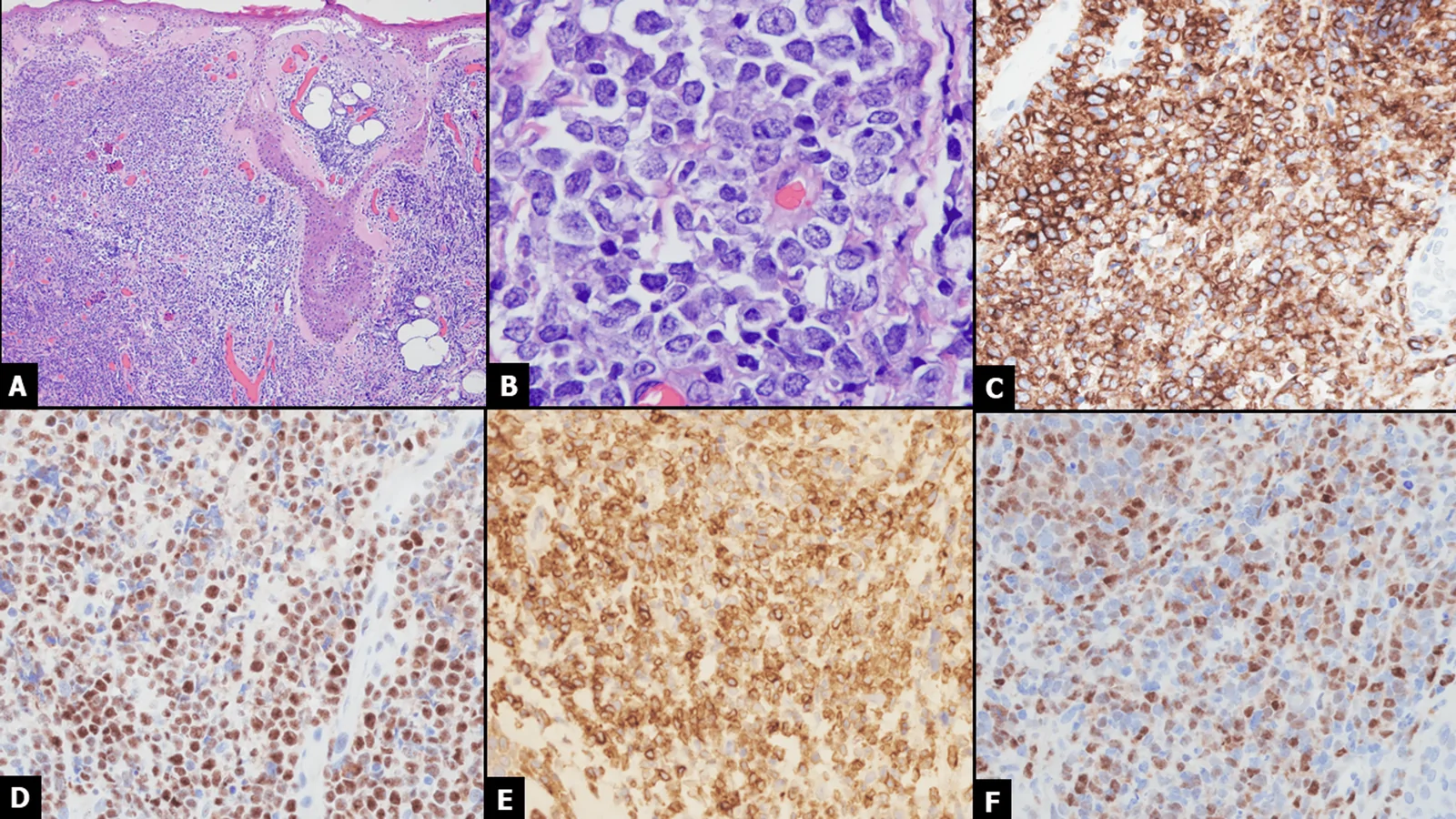
Primary cutaneous diffuse large B-cell lymphoma, leg type. (A) At low magnification, the neoplasm diffusely replaces the dermis without involvement of the epidermis (hematoxylin-eosin, 100×). (B) The neoplastic cells are large (hematoxylin-eosin, 1000×). (C-F) Immunohistochemical analysis shows that the neoplastic cells are positive for CD79a (C), FOXP1 (D), BCL2 (E), and MUM1/IRF4 (F) (immunohistochemistry with haematoxylin counterstain, 400×).

The lesion was erythematous, raised, and increasingly friable during the two weeks prior to initially presenting to her local provider. A skin shave biopsy was performed, revealing diffuse large B-cell lymphoma with a non-germinal center B-cell immunophenotype. The neoplasm diffusely involved the dermis, without epidermotropism, and was composed predominantly of centroblasts (Figures 2A, 2B). Immunohistochemical analysis showed that the lymphoma cells were positive for CD20, CD79a (Figure 2C), FOXP1 (Figure 2D), BCL2 (Figure 2E), BCL6 (variable), and MUM1/IRF4 (weak) (Figure 2F), and were negative for CD3, CD10, and MYC (weak, <40%). The Ki-67 antibody showed a proliferation rate of 60%-70%. Fluorescence in situ hybridization analysis was negative for MYC rearrangement. The location and morphology suggested PCDLBCL-LT. The patient declined a bone marrow biopsy because she was not pursuing systemic therapy due to her age, fragility, and transportation issues.

The initial positron emission tomography/computed tomography (PET/CT) scan (Figure 1B) showed two hypermetabolic cutaneous/subcutaneous lesions in the left distal leg, consistent with the biopsy findings. The larger lesion measured 1.5 × 2.8 cm (standardized uptake value (SUV) of 10.6), and the left lateral leg lesion measured 0.8 × 1.2 cm (SUV of 8.7). Degenerative changes were observed in the shoulders, spine, and hips. No other active disease sites were present. The patient had stage II disease per Ann Arbor staging because of her two noncontinuous disease sites. Her overall Eastern Cooperative Oncology Group (ECOG) performance status score was 2. Normal laboratory findings and the absence of extracutaneous involvement and systemic symptoms further supported a diagnosis of PCDLBCL-LT. The lesion continued to enlarge rapidly prior to treatment initiation (Figure 1C).

**Figure 3:**
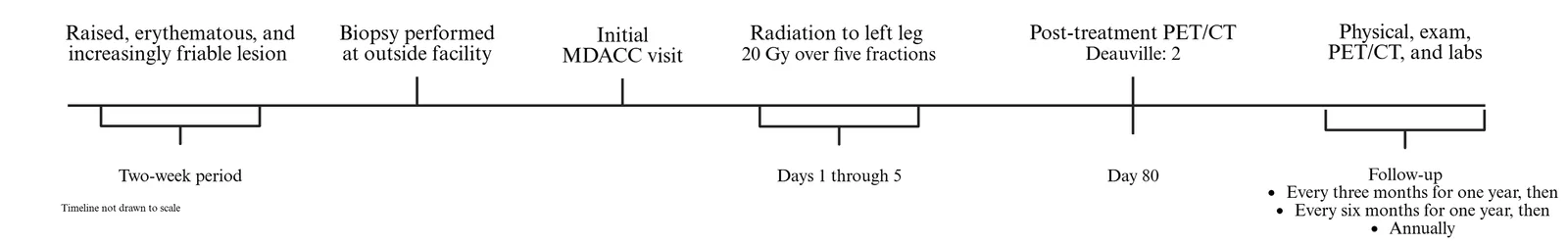
Chronological timeline of disease diagnosis, treatment, and follow-up. Deauville: Deauville score; Gy: gray; MDACC: University of Texas MD Anderson Cancer Center; PET/CT: positron emission tomography/computed tomography

The patient received a total of 20 gray (Gy) over five fractions to her left ankle. She declined any systemic therapy due to her concerns about her age and fragility, as stated above. Furthermore, she had a shortened course of radiation therapy due to transportation difficulties related to her limited independent functioning. The patient submitted images seven days post-radiation therapy, demonstrating flattening of the treated lesions (Figure 1D, left). The lesion displayed some erythema and hyperpigmentation, which were expected skin reactions given the hypofractionated course. The PET/CT scan completed at the end of treatment (three months post-radiation) demonstrated a complete metabolic response with a Deauville score of 2 (Figure 1E). After achieving complete response (CR), the patient was seen every three months for PET/CT and laboratory testing during the first year (Figure 3). She was seen every six months during the second year and is now seen annually. The patient remains without evidence of disease five years later (Figure 1D, right).

## Discussion

PCDLBCL-LT remains a distinct entity within the large B-cell lymphoma category in both the ICC and WHO-EORTC classification systems but is challenging to diagnose due to its overlapping clinical and histopathological features with other skin conditions [6,10,11]. Histopathological and immunophenotypic evaluation, as well as clinical correlation, are essential to distinguish PCDLBCL-LT from other CBCLs [6]. This distinction is important because of the aggressive nature of PCDLBCL-LT, which requires prompt diagnosis and initiation of appropriate treatment. An analysis of 967 PCDLBCL-LT patients reported a median overall survival (OS) of 42 months [14]. The five-year OS for PCDLBCL-LT is between 50% and 60%, and the five-year disease-free survival (DFS) is much lower (approximately 20% to 40%) [7,15]. Given this, the five-year OS and DFS achieved in our patient are remarkable, particularly given her several negative prognostic factors (leg localization, age older than 75 years, and multiple lesions), each of which is associated with worse outcomes [3,8].

Rituximab, cyclophosphamide, doxorubicin, vincristine, and prednisone (i.e., R-CHOP), with or without local ISRT, was the standard treatment for PCDLBCL-LT when our patient presented for treatment [4]. NCCN guidelines have since been updated such that Pola-R-CHP and local ISRT are recommended as first-line therapy for solitary regional disease, with local ISRT alone reserved for patients unable to tolerate chemoimmunotherapy [12]. Radiation monotherapy, such as local ISRT, is a particularly attractive treatment approach for elderly patients, the primary patient population with PCDLBCL-LT, because it is less intensive. In the previously mentioned analysis of 967 PCDLBCL-LT patients, there was no statistically significant difference in median OS between patients receiving chemotherapy (41 months) and radiation monotherapy (39 months) [14]. Further, in a retrospective analysis of 20 PCDLBCL-LT patients, all achieved a CR with radiation monotherapy, compared with 75% among patients treated with the physician’s choice for first-line treatment [13]. The high CR rate achieved with radiation monotherapy makes sense because CBCLs are highly sensitive to radiation. Lymphoma cells undergo rapid apoptosis in response to relatively low doses of radiation and, unlike normal, healthy cells, lack DNA repair capacity [16]. Furthermore, because PCDLBCL-LT lesions are superficial, radiation can be delivered precisely, allowing nearly all irradiated lesions to achieve CR [13].

While radiation monotherapy provides good local disease control, prior work suggests that it may be insufficient as a definitive treatment for PCDLBCL-LT [13]. For example, in the retrospective analysis of eight PCDLBCL-LT patients treated with radiation monotherapy described above, while all eight patients achieved CR, six subsequently progressed, with progression occurring within a median of 12.5 months [13]. It is interesting to note that this was longer than the median time to progression for the physician’s choice group (5.2 months) [13]. Our patient’s durable CR achieved with radiation monotherapy is thus striking, providing support for its potential as a definitive treatment in very particular patients.

Eleven PCDLBCL-LT cases with similar patient profiles to our patient (i.e., elderly patients) were selected from the published literature to demonstrate the different treatment approaches and their associated outcomes (Table 1). The cases also highlight the challenges patients and oncologists face with disease progression, relapse, and resistance to therapy. Interestingly, three patients declined chemotherapy for their treatment due to their age, life expectancy, or comorbidities [5,6]. Compared with the cases presented in Table 1, our patient’s outcome is notably favorable. Many patients in our literature review experienced disease progression, relapse, and/or death within one to three years following treatment completion, regardless of treatment modality.

**Table 1:** PCDLBCL-LT cases and their treatments. A review of published PCDLBCL-LT cases, their treatments, responses, and outcomes. Cases were selected from the published literature to represent the spectrum of treatment approaches and clinical outcomes in PCDLBCL-LT, with emphasis on elderly patients. autoSCT: autologous stem cell transplant; BR: bendamustine, rituximab; CHOP: cyclophosphamide, doxorubicin, vincristine, prednisone; cm: centimeter; CR: complete response; F: female; Gem: gemcitabine; Gy: gray; M: male; mets: metastatic disease; N/A: not reported or available; PCDLBCL-LT: primary cutaneous diffuse large B-cell lymphoma, leg type; PD: progressive disease; PR: partial response; R2: lenalidomide, rituximab; R-CHOP: rituximab, cyclophosphamide, doxorubicin, vincristine, prednisone; R-CVP: rituximab, cyclophosphamide, vincristine, prednisone; R-DHAC: rituximab, dexamethasone, high-dose cytarabine, and carboplatin; R-GemOx: rituximab, gemcitabine, oxaliplatin; RICE: rituximab, ifosfamide, carboplatin, etoposide; RT: radiation therapy; T1a: tumor < 5 cm and cover < 10% of body; T1b: tumor > 5 cm and cover < 10% of body; T2: tumors cover > 10% of body; UTI: urinary tract infection

Age and sex	Lesion size or stage	Treatment	Response	Outcome (duration)	Reference
84M	N/A	R-CVP x 6 cycles, then RT (41.6 Gy); at relapse four years later, BR x 6 cycles, then R2 with PD, then ibrutinib with CR	CR	8+ months	[2]
76M	N/A	R-CHOP, RT (3000 cGy)	CR, 5 months	2+ years	[5]
83M	6 cm x 7 cm	RT (unknown dose)	CR	1.5+ years	[6]
72M	4 cm x 4 cm	RT (unknown dose)	CR	2+ years	[6]
45F	Largest: 10 cm x 11 cm x 19 cm	CHOP	CR	No recurrence at 6 months	[17]
66M	Largest: 6 cm x 5 cm x 2 cm	CHOP	CR	No recurrence at 6 months	[17]
86M	T1b	Mini-R-CHOP; at relapse eight months later, RT with CR for 15 months, then lenalidomide	CR	CR for 38 months, then deceased from UTI/sepsis	[18]
61M	T1b	R-CHOP; at relapse seven months later, cisplatin; at progression eight months later, RICE with CR; at relapse 15 months later, Gem/doxorubicin with autoSCT	Progression	Deceased at 18 months	[18]
81F	T2	Mini-R-CHOP; at relapse 12 months later, pleural effusion and mesenteric relapse	Progression	Deceased at 12 months	[18]
73F	T1a	RT (32 Gy); at relapse 11 months later, R-CHOP with CR; at relapse 22 months later, R-DHAC	Progression	Deceased at 40 months	[18]
84F	T1a	R-CHOP, then progression with bone involvement; R-GemOx with PD; RT (36 Gy) with PD with pulmonary mets, R2 progression	Progression	Deceased at 9 months	[18]

Conversely, the two patients in Table 1 and our patient who were treated with radiation monotherapy all achieved CR [6]. Further, the two patients maintained their disease-free status at their respective follow-up appointments of 1.5 and 2 years, and our patient remained disease-free for at least five years; longer follow-ups were not reported for the two patients in Table 1 [6]. Our case adds to the literature by demonstrating that radiation monotherapy can achieve and sustain CR in a PCDLBCL-LT patient, even one with multiple adverse prognostic factors.

Altogether, these cases support the use of individualized strategies to accommodate patient wishes while effectively treating their malignancy. Regular follow-up to ensure adherence to medical advice is crucial for monitoring disease progression and guiding appropriate interventions. Lastly, multidisciplinary collaboration among oncologists, dermatologists, pathologists, radiation specialists, and all healthcare professionals is necessary to improve patient outcomes [6].

## Conclusions

PCDLBCL-LT represents a rare and aggressive type of extranodal non-Hodgkin lymphoma. The clinical course of affected patients underscores the importance of early yet accurate diagnosis through histopathological evaluation and immunophenotypic analysis. This case demonstrates that definitive radiation monotherapy can achieve durable local control and long-term remission in selected elderly patients with localized PCDLBCL-LT who are not candidates for systemic therapy. Individualized treatment planning and multidisciplinary care remain crucial.

Despite advances in treatment, disparities in access to care and the rarity of PCDLBCL-LT pose challenges for its timely diagnosis and optimal management. Multidisciplinary care is essential for optimizing outcomes for patients with PCDLBCL-LT. Moreover, long-term follow-up remains necessary to monitor for disease recurrence and late treatment effects. The case we report highlights the importance of an individualized approach to treating rare types of lymphoma, leading to optimized patient outcomes and improvement in quality of life.
